# A combined approach of robot-assisted laparoscopic pyeloplasty and flexible endoscopy to treat concomitant ureteropelvic junction obstruction and calyceal stones in children: Technical considerations and review of the literature

**DOI:** 10.3389/fped.2022.1017722

**Published:** 2022-10-28

**Authors:** Yuenshan Sammi Wong, Ka Lun Lo, Kristine Kit Yi Pang, Yuk Him Tam

**Affiliations:** ^1^ Department of Surgery, Division of Paediatric Surgery & Paediatric Urology, Prince of Wales Hospital, The Chinese University of Hong Kong, Hong Kong, China; ^2^ Department of Surgery, Hong Kong Children’s Hospital, Hong Kong, China; ^3^ Department of Surgery, Division of Urology, Prince of Wales Hospital, The Chinese University of Hong Kong, Hong Kong, China

**Keywords:** robot-assisted laparoscopic pyeloplasty, flexible endoscopy, ureteropelvic junction obstruction, renal stones, calyceal stones

## Abstract

The management of children with concomitant ureteropelvic junction (UPJ) obstruction and calyceal stones remains challenging. The various treatment options available for pediatric nephrolithiasis may require multiple sessions, and the techniques by themselves are not designed for simultaneous correction of UPJ obstruction. Recently, success in combining robot-assisted laparoscopic pyeloplasty (RALP) and flexible endoscopy has been reported by multi-institutional studies to treat children with concomitant UPJ obstruction and renal stones. Given the paucity of technical details of this novel approach in the existing literature, we herein report our techniques to treat two girls aged 6 and 10 years who had concomitant UPJ obstruction and multiple stones in mid- and lower poles calyces. Three robotic ports were used without any assistant ports. A flexible endoscope, either a cystoscope or a single-use ureteroscope, was introduced *via* the undocked epigastric port to perform nephroscopy and stones removal after the renal pelvis was opened. The rest of the RALP was completed in the usual manner. Technical modifications were employed to facilitate the flexible endoscope to examine the entire calyceal system. Both patients underwent successful surgical procedures by the combined approach without any intra- or post-operative complications. Three and 14 stones were removed from each of the patients respectively. Postoperative investigations demonstrated successful correction of UPJ obstruction and complete stone clearance in both patients. A combined approach of RALP and flexible endoscopy is a safe and effective technique to treat concurrent UPJ obstruction and calyceal stones in children.

## Introduction

There are a variety of techniques which are established as first-line treatment modalities for urolithiasis in children (1–4). However, such endourological and percutaneous techniques are not designed to work simultaneously with other surgical techniques when renal stones develop concomitantly with other urinary tract pathologies that require surgical treatment. Ureteropelvic junction (UPJ) obstruction is the most common type of obstructive pathology to the upper urinary tract requiring surgical intervention in children. In adults, the incidence of UPJ obstruction with concomitant renal stones has been reported to be around 20% (5). The incidence in children is believed to be much lower. Recent international guidelines include the options of open surgery or minimally invasive surgery (MIS) for the simultaneous correction of UPJ obstruction and stones removal, with the potential advantage of avoiding repeated general anesthesia in affected children (3, 6).

Despite its utilization in children for more than two decades, laparoscopic pyeloplasty has never gained popularity due to its technical challenges. In contrast, robot-assisted laparoscopic pyeloplasty (RALP) has become a popular MIS technique in correcting UPJ obstruction in children and is the most commonly performed robotic surgery in the field of pediatric urology (7, 8). Not surprisingly, robotic technology has also been explored in recent years to manage urolithiasis in children (6, 9, 10). While the concurrent retrieval of stones in the renal pelvis may appear straightforward at the time of pyeloplasty, extracting calyceal stones remains challenging. Three pediatric studies have recently reported their successful experience of combining RALP and the use of a flexible endoscope to treat concomitant UPJ obstruction and nephrolithiasis (9–11). Although the evidence remains limited in the existing literature, such combined approach provides a very promising way to treat two urological conditions concurrently by MIS.

We herein report our single-institution experience of using the combined endoscopic and robot-assisted approach to treat concomitant UPJ obstruction and calyceal stones in two children. We aimed to describe the technical modifications we had employed in the setting of using only three ports and highlight how the technical modifications facilitated the endoscopic examination of the calyceal system to enhance stone clearance.

## Materials and methods

### Patients

Approval was obtained from the clinical research ethics committee of our institution to retrospectively review the medical records of children who underwent RALP at our center. Both of the patients reported in this study were girls. They were aged 6 and 10 years at the time of surgery, with a body weight of 19 and 26 kg respectively. Prior to surgery, both patients had undergone extensive radiological investigations including serial ultrasounds, diuretic renal radioisotope scan, KUB plain x-ray, and non-contrast computed tomography (CT) scan. Left UPJ obstruction was diagnosed in both patients who had grade 4 hydronephrosis in their left kidneys (as per the Society of Fetal Urology criteria) with diffuse parenchymal thinning and obstructed drainage in radioisotope scans with diuretic t-half >20 min. The anteroposterior diameters (APD) of the renal pelvis were 17 and 28 mm, respectively. The left kidney differential renal function values were 45% and 46%, respectively. Multiple renal stones ranging from 4 to 8 mm were demonstrated in the mid- and lower pole calyces of the left hydronephrotic kidney in both patients by ultrasound, plain x-ray, and non-contrast CT scan. Both patients underwent the combined approach of RALP and flexible endoscopy to remove the calyceal stones.

### Surgical techniques

**Figure 1:**
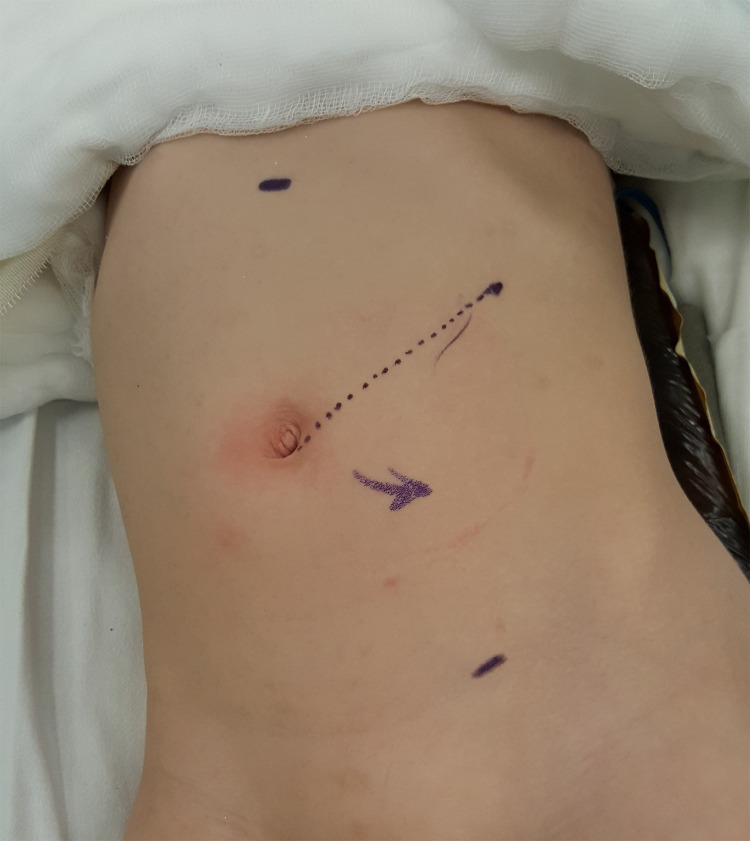
Our typical port sites for left-sided RALP with the camera port at infraumbilical ring and two 8-mm working ports at epigastrium and left iliac fossa mid-clavicular line.

**Figure 2:**
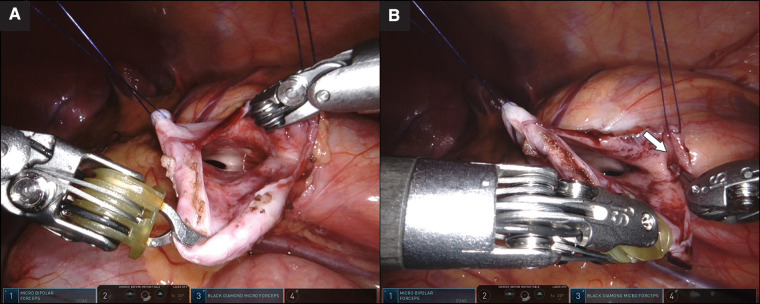
(**A**) Wide exposure of the pelvicalyceal system with two transabdominal hitch sutures. (**B**) Lower pole calyx with narrow opening (arrow) identified under the laparoscopic view with the assistance of robotic instrument.

**Figure 3:**
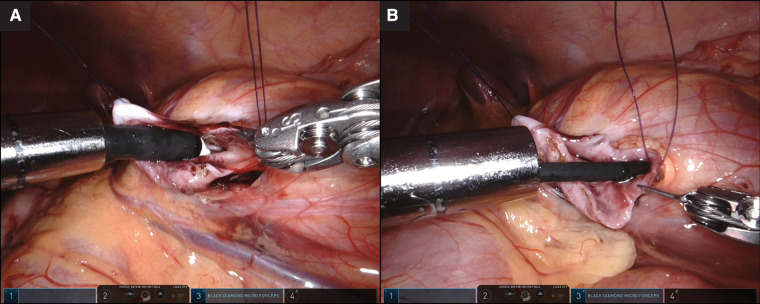
(**A**) Easy access by a Fr16 flexible cystoscope to the upper and mid-pole calyces for examination. (**B**) The lower pole calyx with narrow infundibulum allowed the passage of a Fr8.7/9.3 flexible ureteroscope after failed attempts by the flexible cystoscope. (**A,B**) Tip of the epigastric port close to the pyelotomy to facilitate the manipulation of the flexible scope.

We have previously reported our techniques of performing RALP in children (12). In both patients, the da Vinci Xi model (Intuitive Surgical, Sunnyvale, CA) was used. We routinely used three 8-mm ports for pediatric RALP without any assistant ports *via* a transperitoneal approach. An 8-mm camera port was inserted along the infraumbilical ring by open technique. Two 8-mm working ports were placed at the epigastrium and the left iliac fossa at the mid-clavicular line (Figure 1). Flexible endoscopy was performed after the renal pelvis was opened. Figures 2, 3 illustrate the technical modifications we used for these two patients: (1) Two transabdominal hitch sutures were placed to keep the renal pelvis widely opened after pyelotomy; (2) The robotic arm was undocked at the epigastric port; (3) The epigastric port was advanced until its tip was close to the opened renal pelvis; (4) Nephroscopy was performed by passing a Fr16 flexible cystoscope directly into the renal pelvis *via* the undocked epigastric port. The calyceal system was carefully examined and stones were removed by basket. In the case of a narrow infundibulum inhibiting the passage of the flexible cystoscope, a single-use Fr8.7/9.3 flexible ureteroscope was used. The remaining robotic working arm assisted the flexible nephroscopy by enhancing the exposure of the individual calyces and providing counter traction if necessary. After the completion of nephroscopy and stones extraction, the epigastric port was redocked and RALP continued in our usual manner. A double J stent was inserted in both patients percutaneously over guidewire which was introduced *via* an angiocatheter following our standard technique (12). No abdominal drain was placed in both patients.

## Results

Both patients underwent the combined approach successfully without any intraoperative or postoperative complications. There was no conversion to open surgery or requirement of an additional port. In both cases the left kidneys were in orthotopic positions and no aberrant crossing vessels were identified. In the 10-year-old patient, the flexible cystoscope was able to achieve complete examination of the entire calyceal system as well as stones removal. However, the 6-year-old girl had a narrow infundibulum of the lower pole calyx of her left kidney which inhibited the passage of the cystoscope. A single-use Fr8.7/9.3 flexible ureteroscope was subsequently used to complete the examination of the lower pole calyx and removal of the lower pole calyceal stones. A total of three and 14 stones were removed from the mid- and lower pole calyces of each patient, respectively. The operative times from skin incision to wound closure were 225 and 232 min, respectively. Both patients had a hospital stay of 2 days. The double J stent was removed in 4 weeks in both patients.

Postoperative investigations included a diuretic radioisotope scan, KUB plain x-ray, and ultrasound examination at 1–2 months and 6–9 months. At a follow-up of 9 and 11 months, successful pyeloplasty was confirmed in both patients by ultrasound findings showing resolution of hydronephrosis with the APDs reduced to 3 and 5 mm, respectively. Postoperative radioisotope scans showed improved drainage of their left kidneys with the diuretic t-half values being 15 and 12 min and left differential renal function being 45% and 53%, respectively. Complete stone clearance was demonstrated in both patients by ultrasound and plain x-ray. Stone analysis showed calcium oxalate in both patients. One of the patients had a complete genetic workup which did not identify any clinically significant variants.

## Discussion

The increase in the global incidence of pediatric urolithiasis over the past few decades has led to an increasing demand for surgical interventions and the progressive development of treatment modalities appropriate for children (1, 13, 14). Currently, a variety of treatment options such as percutaneous nephrolithotomy, ureteroscopy, retrograde intrarenal surgery, and extracorporeal shock wave lithotripsy are available for children affected by urolithiasis (1, 4, 15). Stone formation in the urinary tract involves the complex interaction of multiple factors, and obstruction is a known predisposing factor that needs to be excluded. UPJ obstruction with concomitant renal calculi is uncommonly seen in children, and it poses a special challenge to management. Performing the correction of UPJ obstruction and stone extraction in separate procedures is an option, but this is not a preferred strategy in children as it would inevitably require multiple exposures to general anesthesia.

There is emerging evidence in adult studies which demonstrate the safety and effectiveness of simultaneous correction of UPJ obstruction and removal of renal calculi *via* robot-assisted laparoscopic surgery with concurrent nephroscopy by flexible or rigid scope for stones extraction (5, 16–18). Atug et al. in 2005 was the first to report using the combined approach of RALP and flexible endoscopy in adult patients (16). This combined surgical strategy can be applied similarly to pure laparoscopy (19, 20). However, the technical difficulties of performing pyeloplasty by pure laparoscopy in children have been unequivocally recognized. In contrast, RALP has become the most popular MIS modality to correct pediatric UPJ obstruction in western countries given its technical advantages and improved ergonomics (7). A recent systematic review has found evidence demonstrating excellent outcomes of RALP in primary pyeloplasty in pediatric populations, providing evidence to support its use in challenging scenarios such as redo pyeloplasty, UPJ obstruction in horseshoe kidneys, and UPJ obstruction with concomitant renal stones (21).

A literature search was performed and five articles were identified which reported experience in using the combined approach of RALP and flexible endoscopy to treat concurrent UPJ obstruction and renal calculi in children (Table 1). These include two multi-institutional case series (9, 10), two single-institution case series (11, 22), and one case report (23). Lee et al. was the first to report the successful management of concomitant UPJ obstruction and renal stone in an adolescent by the combined approach of RALP with flexible nephroscopy (22). The two multi-institutional studies reported by Esposito et al. (9) and Roth et al. (10) included 11 and 21 children managed in four and five institutions, respectively. A 100% success rate in the correction of UPJ obstruction was reported in one of the studies (9). Taking into account other patients without concomitant UPJ obstruction who were included in the studies, the two studies reported stone-free rates of 70%–80% after the initial surgery using the combined approach of robot-assisted laparoscopy and flexible endoscopy (9, 10). Furthermore, Masieri et al. reported the largest single-institution series of seven children at a median age of 7 years who had concomitant UPJ obstruction and renal stones (11). The authors reported a 100% success rate in the concurrent correction of UPJ obstruction and stone extraction using the combined approach of RALP and flexible endoscopy (11). Two studies also reported using laser lithotripsy *via* flexible nephroscopy to fragment the stones where they were too big to be directly removed from the calyces (10, 23). In the absence of concomitant UPJ obstruction, robot-assisted pyelolithotomy combined with flexible nephroscopy has been reported as a viable option to manage complex and challenging cases of nephrolithiasis in both adult (24–26) and pediatric populations (9, 10, 22) where other standard modalities may not be feasible due to altered anatomy, where the stone burden is large, or when multiple prior procedures have been attempted but failed. The concurrent flexible endoscopy offers the advantages to examine individual calyces, to extract calyceal stones which cannot be reached by robotic instruments, and to introduce energy source for stone fragmentation if deemed necessary.

**Table 1:** Summary of pediatric literature reporting on the combined approach of RALP and flexible endoscopy to treat concomitant UPJ obstruction and renal stones.

Authors, Year	Study design	Number of patients with concomitant UPJ obstruction and renal stones, and patients’ demographics	Technical highlights	Surgical outcomes
Lee et al., 2007 (22)	Single-center case series including patients without concomitant UPJ obstruction	1 patient ^a^Mean age of 16.6 years ^a^Mean BW of 53 kg	3 ports Flexible nephroscopy by cystoscope Stones extraction by robotic instruments or by graspers or basket *via* flexible endoscopy Double J stent No abdominal drain	Stone-free rate 100% ^a^Stone-free rate 75% after initial surgery
Roth et al., 2020 (10)	Multi-institutional case series including patients without concomitant UPJ obstruction	21 patients ^a^Median age at 12.2 years ^a^Median BMI at 17.5 kg/m^2^	4 ports by traditional or HIdES approach Stones extraction by robotic instruments or by graspers or basket *via* flexible endoscopy Laser lithotripsy if indicated Double J stent	^a^Stone-free rate 70.4% after initial surgery
Scarcella et al., 2020 (23)	Single-center case report	1 patient 4-year-old BW 24 kg	4 ports + percutaneous liver retractor Flexible nephroscopy by Fr7.7/9.5 single-use ureteroscope Stone extraction by laser lithotripsy and basket *via* flexible endoscopy Double J stent Abdominal drain	Stone-free rate 100% Pyeloplasty success rate 100%
Esposito et al., 2021 (9)	Multi-institutional case series including patients without concomitant UPJ obstruction	11 patients ^a^Median age at 8.5 years ^a^Medan weight 32.2 kg	4 ports, Flexible nephroscopy by ureteroscope Stones extraction by robotic instruments or by graspers or basket *via* flexible endoscopy Double J stent Abdominal drain in some patients	^a^Stone-free rate 80% after initial surgery Pyeloplasty success rate 100%
Masieri et al., 2022 (11)	Single-center case series	7 patients Median age at 7 years Median BMI at 19.6 kg/m^2^	4 ports, Flexible nephroscopy by ureteroscope Stones extraction by robotic instruments or by graspers or basket *via* flexible endoscopy Double J stent Abdominal drain in 3/7 patients	Stone-free rate 100% Pyeloplasty success rate 100%
Present study	Single-center case series	2 patients Aged 6 & 10 years BW 19 & 26 kg	3 ports Long pyelotomy with two transabdominal hitch sutures on renal pelvis Flexible nephroscopy by cystoscope and Fr8.7/9.3 single-use ureteroscope Stones extraction by basket *via* flexible endoscopy Double J stent No abdominal drain	Stone-free rate 100% Pyeloplasty success rate 100%

HIdES, hidden incision endoscopic surgery; BW, body weight; BMI, body mass index.

^a^ Overall results including study subjects without concomitant UPJ obstruction who underwent robot-assisted pyelolithotomy and flexible nephroscopy.

Depending on the surgeon's preference, RALP in children can be performed using three ports (12, 27, 28) or four ports which include three robotic and one assistant port (29–31). The existing literature has illustrated the techniques of using four ports or four ports with an extra percutaneous liver retractor in the combined approach of robot-assisted laparoscopy and flexible nephroscopy in children (9–11, 23). As we routinely perform RALP on infants to adolescents using three ports only (12, 32), we decided to incorporate flexible nephroscopy into our standard techniques for pediatric RALP without adding extra ports. A complete and thorough examination of the entire calyceal system is critical to stone clearance. We found that the technical modifications we employed were helpful to achieve this goal.

The epigastric port provides the best access route for flexible nephroscopy given its position relative to the renal pelvis. In addition to undocking the epigastric port temporarily, we advanced the port such that its tip was close to the pyelotomy. By manually adjusting the direction of the undocked port, the advancement and manipulation of the flexible endoscope during nephroscopy was facilitated with the support of the port at the distal part of the flexible endoscope. We routinely reduce the redundant renal pelvis in pediatric RALP with a long pyelotomy. For the two patients in this report, we decided to insert two transabdominal hitch sutures to elevate the anterior wall of the renal pelvis instead of using just one hitch suture as per our standard RALP. A wide exposure of the pelvicalyceal system, which could be visualized with the laparoscopic view, highlighted the locations of the calyces and provided guidance for the flexible endoscope. The remaining robotic instrument helped to locate any inconspicuous calyceal openings and assisted the entry of the flexible scope into individual calyces. Such technical modifications minimized the amount of irrigation needed to distend the system for evaluation during flexible nephroscopy. Adult studies have reported the use of robotic instruments to seal the pyelotomy during nephroscopy to reduce fluid leak and to distend the system for optimal endoscopic evaluation (5, 17). In contrast, we found that a well-exposed pelvicalyceal system best facilitated flexible nephroscopy by providing a clear road map.

With regard to the choice of flexible endoscope, we consider flexible cystoscope the preferred endoscope provided that the anatomy of the entire pelvicalyceal system allows for its examination. With its outer diameter just above 5 mm, there would be no air leak when the flexible cystoscope is introduced through the 8-mm robotic port. The flexible cystoscope also provides a better endoscopic view than the ureteroscope and is more versatile in case other endourological technologies such as laser lithotripsy are needed. A flexible ureteroscope, however, must be readily available when employing this combined approach as demonstrated in one of our cases that the narrow infundibulum inhibited the entry by the flexible cystoscope. The air leak we encountered during the passage of the flexible ureteroscope *via* the robotic port was controlled by covering the port entry with gauze. The close proximity of the tip of the port and the pyelotomy also reduced the effects of air leak on the flexible endoscope when entering the renal pelvis. Due to our limited experience, we are not certain if there are any cases of pediatric UPJ obstruction with concomitant calyceal stones in which the infundibulum is too narrow to allow the entry of even the smallest flexible ureteroscope available in the market. Nevertheless, it should be noted that it is not just the caliber of the infundibulum that matters. The direction of the endoscope with respect to the calyceal opening, angulation of the endoscope tip, counter traction on the renal pelvis, and adequacy of the exposure of the calyceal opening all contribute to the successful examination of the entire calyceal system.

Technical expertise in performing flexible nephroscopy and endourology in lithotripsy is essential for this combined approach. This may be a limiting factor for pediatric surgeons who may not have enough experience with flexible endoscopy in the urinary tract or other endourological techniques used to manage nephrolithiasis in children. The use of a rigid scope for nephroscopy and stones extraction in the combined approach has been reported in adult patients with concomitant UPJ obstruction and renal calculi (5). The authors reported a 90% stone-free rate in nine patients (5). This technique, however, required the rigid scope to be introduced *via* different ports in order to examine the entire calyceal system. We strongly believe that a flexible endoscope is preferred as only the epigastric port needs to be undocked, and the relatively small pelvicalyceal system in children makes a complete examination of the calyces by a rigid scope difficult, if not impossible. We operated on the two patients jointly with an adult urologist who performed the flexible endoscopy and stones retrieval. In our locality, pediatric nephrolithiasis requiring interventions is quite uncommon and even tertiary institutions have limited experience with intrarenal surgery for nephrolithiasis in children. Although no single surgical team at our institution could stand alone for this combined approach, the combined expertise of both pediatric and adult surgeons provided a solution.

Robot-assisted laparoscopy has emerged as a new surgical alternative for select children affected by urolithiasis. Although data remains limited in the literature, children with concomitant UPJ obstruction and renal calculi appear to represent a select group with a strong indication for applying the robot-assisted approach. Combining RALP with flexible nephroscopy can simultaneously correct UPJ obstruction and remove renal calculi in children. This combined approach avoids the need for multiple exposures to general anesthesia and has the potential advantage of reducing the morbidity associated with multiple access routes to the kidneys. We and others have demonstrated its safety and effectiveness using three or four ports. With an established robotic surgery service in pediatric urology, and with the availability of appropriate flexible endoscopes and expertise in using them, this combined approach can be readily adopted to manage children with concomitant UPJ obstruction and calyceal stones.

## Data Availability

The datasets presented in this article are not readily available due to institutional legislation. Further enquiries should be directed to the corresponding author.
